# Impact of the COVID-19 pandemic on malaria cases in health facilities in northern Ghana: a retrospective analysis of routine surveillance data

**DOI:** 10.1186/s12936-022-04154-1

**Published:** 2022-05-15

**Authors:** Anna-Katharina Heuschen, Alhassan Abdul-Mumin, Martin Adokiya, Guangyu Lu, Albrecht Jahn, Oliver Razum, Volker Winkler, Olaf Müller

**Affiliations:** 1 Institute of Global Health, Medical School, Ruprecht-Karls-University, Heidelberg, Germany; 2 Department of Pediatrics and Child Health, School of Medicine, University for Development Studies, Tamale, Ghana; 3 Department of Epidemiology, Biostatistics and Disease Control, School of Public Health, University for Development Studies, Tamale, Ghana; 4 School of Public Health, Medical School, Yangzhou University, Yangzhou, China; 5 Department of Epidemiology and International Public Health, School of Public Health, Bielefeld University, Bielefeld, Germany

**Keywords:** COVID-19, Pandemic, Malaria, Sub-Saharan Africa, Ghana, Northern Region, Health information system, Surveillance, Morbidity, Routine data

## Abstract

**Background:**

The COVID-19 pandemic and its collateral damage severely impact health systems globally and risk to worsen the malaria situation in endemic countries. Malaria is a leading cause of morbidity and mortality in Ghana. This study aims to describe the potential effects of the COVID-19 pandemic on malaria cases observed in health facilities in the Northern Region of Ghana.

**Methods:**

Monthly routine data from the District Health Information Management System II (DHIMS2) of the Northern Region of Ghana were analysed. Overall outpatient department visits (OPD) and malaria case rates from the years 2015–2019 were compared to the corresponding data of the year 2020.

**Results:**

Compared to the corresponding periods of the years 2015–2019, overall visits and malaria cases in paediatric and adult OPDs in northern Ghana decreased in March and April 2020, when major movement and social restrictions were implemented in response to the pandemic. Cases slightly rebounded afterwards in 2020, but stayed below the average of the previous years. Malaria data from inpatient departments showed a similar but more pronounced trend when compared to OPDs. In pregnant women, however, malaria cases in OPDs increased after the first COVID-19 wave.

**Conclusions:**

The findings from this study show that the COVID-19 pandemic affects the malaria burden in health facilities of northern Ghana, with declines in inpatient and outpatient rates except for pregnant women. They may have experienced reduced access to insecticide-treated nets and intermittent preventive malaria treatment in pregnancy, resulting in subsequent higher malaria morbidity. Further data, particularly from community-based studies and ideally complemented by qualitative research, are needed to fully determine the impact of the pandemic on the malaria situation in Africa.

## Background

Malaria remains one of the leading causes of morbidity and mortality in sub-Saharan Africa (SSA). Globally, there have been 627.000 malaria related deaths in 2020, 12% more than in 2019; 68% of these additional deaths were attributed to indirect consequences of the COVID-19 pandemic [1]. In Ghana, malaria is responsible for 10% of the overall mortality and nearly one quarter of all under five childhood deaths [1, 2]. The 2015–2020 Ghana Strategic Action Plan aimed to reduce the burden of malaria by 75.0% [3], but the COVID-19 pandemic could halt or even reverse the declining trends. In 2020, malaria was the cause of more than one third of all OPD attendances [4]; moreover 17.6% of OPD visits of pregnant women were due to malaria [5].

The global spread of the coronavirus disease 2019 (COVID-19) was declared a *Public Health Emergency of International Concern* at the end of January 2020 [6]. Many African governments responded rapidly to this threat by implementing control measures even before first cases were detected in their countries, comprising border closures, movement restrictions, social distancing and school closures [7]. SSA accounted for only about 3.5% of the globally reported COVID-19 morbidity and mortality, mostly from the southern and northern rims of the continent, while being home to 17% of the global population [8]. This may be explained by factors such as a younger population, hotter climate, interferences with other infectious diseases, but especially lack of diagnostics and underreporting [9, 10]. Ghana was among the countries with the highest reported COVID-19 cases and deaths in western and central SSA, as of the end of 2020 [11]. COVID-19 vaccinations started in February 2021 but coverage in Ghana is still low with only 10% of the population fully vaccinated by February 2022 [12].

The socio-economic disruptions associated with the disease and the preventive measures present huge challenges for health systems and whole societies, especially in low- and middle income countries [13]. In the highly malaria-endemic African countries, the progress made in malaria control during the last two decades was feared to be reversed by the side effects of the COVID-19 pandemic [14, 15]. In the Ghanaian context, as no ITN mass campaigns were scheduled for 2020, the worst-case scenario presented in a modelling study by Weiss et al. could have been a decline in access to anti-malarial medication by 75%, resulting in an increase of malaria morbidity and mortality by 13% and 55%, respectively [14]. Overall, the predicted public health relevant effects of the COVID-19 pandemic on malaria include shared clinical disease manifestations leading to diagnostic challenges, shortages of anti-malarial medication, rapid diagnostic tests, preventive tools and personal protective equipment, decreasing quality of surveillance systems, and re-allocation of funds and professionals towards COVID-19 control activities [16].

This study aims to describe potential effects of the COVID-19 pandemic on malaria cases in health facilities in the Northern Region of Ghana – a highly malaria endemic region. The research hypothesis that “lower access to health care services in combination with impaired malaria surveillance systems may have led to a lower number of reported malaria cases in highly endemic countries” will be investigated [16]. This hypothesis leads to the specific research question if the COVID-19 pandemic has led to less reported malaria cases in northern Ghana in 2020.

## Methods

### Study area

Ghana, with its population of about 31 million, lies in western SSA and has a relatively well functioning health care system [17, 18]. The Northern Region, with its capital city Tamale, had a population of 1.9 million in 2020. The socio-economic situation of the Northern Region is below the national average of the country and the region has the highest mortality rate in children under the age of five years [19].

Malaria is highly endemic in northern Ghana with a seasonal transmission pattern that is strongest between July and November. ITNs are a major malaria prevention strategy in Ghana; in 2019, about 74% of households owned at least one ITN and 52% of all households had at least one ITN per two people [20]. According to the state of the nation’s health report 2018, the malaria prevalence in children under- five years in the Northern Region is 40%, the highest in Ghana [21]. Regarding the epidemiology of COVID-19, Ghana recorded the first cases in March 2020. The government responded immediately with the implementation of social gathering and travel restrictions as well as school closures. The country’s major cities were placed under partial lockdown soon after. This lockdown started on March 30, 2020, and lasted for two weeks. Schools were partially reopened on June 21, 2020, and borders were reopened to international airlines on September 21, 2020 [22]. As at end of March 2021, the country had a total of 90,583 confirmed cases and 743 deaths [23]. These cases were clustered around two major waves in March-September 2020 and January-March 2021. The first wave coincided partly with the rainy season in the Northern Region, the time when the majority of cases of malaria in children and pregnant women are recorded [24].

In Ghana, effects of the COVID-19 pandemic on malaria control interventions concerned the country’s stock of artemisinin-based combination therapy, the functioning of its insecticide-treated mosquito net (ITN) routine distribution, and the overall access to primary health care services and facilities [25].

Although the lockdown did not include the Northern Region directly, the other pandemic control measures including the ‘stay at home unless absolutely necessary‘ campaign, suspension of OPD services in many hospitals and the general anxiety among the population, led to reduction in antenatal and child welfare clinic attendance. This situation could have further affected malaria control measures as the antenatal and child welfare clinics are two major service delivery points where education on malaria prevention, intermittent preventive treatment in pregnancy (IPTp) services and distribution of ITNs to children and pregnant women are carried out [26].

## Study design and data

This retrospective observational study uses monthly malaria morbidity data on the overall number of outpatients (interpreted as less severe cases) and inpatients (more severe cases). Additionally, all outpatient visits (including non-malaria related visits) were analysed. Subgroup analysis was performed for the two groups at high risk for severe malaria manifestations, children under five years of age and pregnant women [1]. Cases were extracted from the *District Health Information Management System II* (DHIMS2) on demographic and health parameters of northern Ghana from January 1, 2015, to December 31, 2020. This system was implemented in 2007 with an update in 2012 and has improved the data quality and completeness since [27].

Malaria diagnosis was based either on the results of rapid diagnostic tests or microscopy.

Mid-year population estimates of the Northern Region of Ghana were also provided through the DHIMS2.

## Analysis

The data have been processed with Microsoft Excel Version 16.52 and analyzed with Stata IC Version 16 (StataCorp, College Station, TX, USA). Monthly rates of OPD visits and confirmed malaria cases for the year 2020 and as a comparison for the years 2015–2019 separately as well as mean rates with 95% confidence intervals (95% CI) have been calculated and plotted using population figures of the Northern Region of Ghana. Additionally, quarterly rates are presented; rates of 2020 versus the combined rates of 2015–2019 have been compared with the z-test. The data allowed analysing children under five years and pregnant women separately using the fraction of the under-five population (14% of the population) and the fraction of women between 15 and 45 years (23% of the population) as estimates of the respective population denominators [28]. Malaria deaths have not been analysed due to low quality of the mortality data sets.

## Results

Table 1 presents a brief description of the dataset. Altogether 5.8 million OPD visits were reported between 2015 and 2020; 39% of those were diagnosed with malaria. Of all malaria cases, 20% were children under the age of five years and 2% were pregnant women. 295,465 patients were hospitalized with diagnosed malaria, 56% of those were children under the age of five. The mean population in the Northern Region of Ghana of the years from 2015 to 2020 was 1,842,701.

**Table 1:** Description of the dataset on outpatients and malaria patients recorded in northern Ghana health facilities during the years 2015–2020

	Total number	Percentage (%)
Outpatient department visits		
All OPD	5,804,910	100
Malaria confirmed	2,278,296	39
Malaria confirmed among children < 5 years	454,779	20
Malaria confirmed among pregnant women	46,693	2
Hospital–admitted patients		
Malaria confirmed	295,465	100
Malaria confirmed among children < 5 years	165,313	56
Mean mid–year population		
Total population	1,842,701	100
Children < 5 years	257,978	14
Women aged 15 to 45	423,821	23

**Fig. 1:**
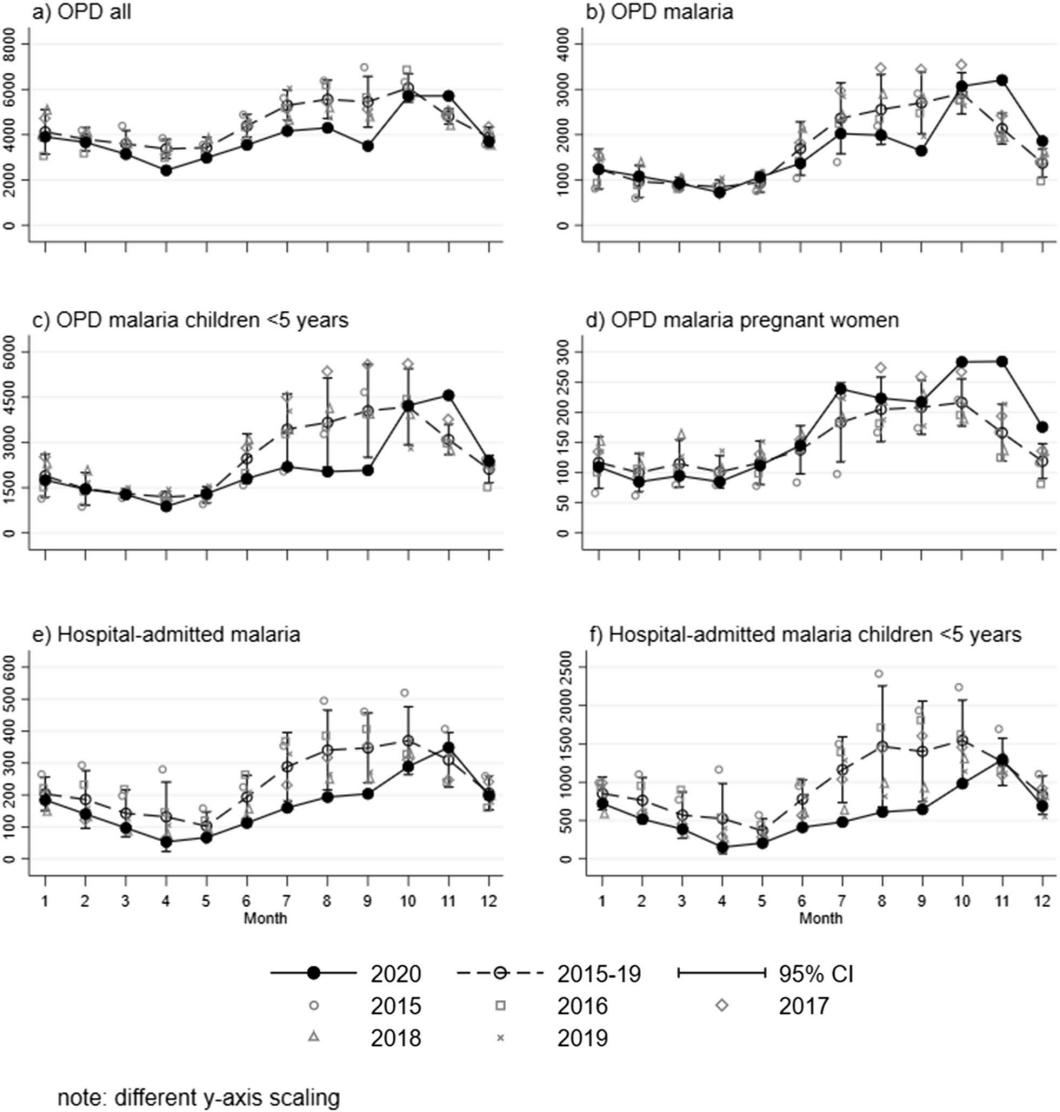
Reported monthly rates (**A** for all outpatient department (OPD) visits per 100,000 of the general population; **B** for OPD visits with confirmed malaria per 100,000 of the general population; **C** for OPD visits with malaria in children under the age of 5 per 100,000 of all children under 5, **D** for OPD visits with malaria in pregnant women per 100,000 of all women aged 15–45, **E** for hospital-admitted patients with malaria per 100,000 of the general population; **F** for hospital-admitted malaria in children under 5 per 100,000 of all children under 5) in health facilities of the Northern Region, Ghana, for the years 2015–2020

Figure 1 presents the case rates of the different outcomes reported from health facilities in the Northern Region of Ghana for the years 2015-20 separately as well as a combined rate for the period 2015 to 2019. All OPD visits (Fig. 1a), including also non-malaria patients, have experienced a decline in March/April 2020 (the months where COVID-19 control measures were implemented in the country) and stayed low during the following months. After a further decrease in September 2020, the numbers increased again in October 2020 to the levels observed in previous years. This trend is similar but not as pronounced in the malaria OPD visits (Fig. 1b). The decline in accessing OPD malaria health care is strongest in children under the age of five years, especially from June to September 2020 (Fig. 1c). In pregnant women, however, a different trend with an increase of malaria cases, starting in June and exceeding previous year levels, can be observed (Fig. 1d). The 2020 numbers of the hospital-admitted malaria patients (March till October) stayed consistently below the numbers from previous years (Fig. 1e); and in accordance with the OPD figures, this trend is more pronounced in children under five years (Fig. 1f).

The rate ratios (RR), depicting quarterly measures comparing the rates of 2020 to the combined rates of the years 2015 to 2019, are presented in Table 2. General OPD visits were significantly reduced in the 2nd and 3rd quarters of 2020 compared to the previous years by up to 27%, with a return to previous standards at the end of the year. For the overall malaria cases only the 3rd quarter of 2020 experienced a decrease by 26%, but increases by 27% in the 4th quarter. Ambulatory malaria cases in children under five experienced stronger reductions compared to previous years by 43% in the 3rd quarter of 2020, and increases by 20% in the 4th quarter. These evolutions are not mirrored by the population of pregnant women with malaria infections, where no major reductions were observed during the first quarters of 2020 compared to previous years but with an earlier and stronger increase that reached 48% in the 4th quarter. The situation is slightly different in patients admitted to the hospital with malaria. The reductions in the 2nd and 3rd quarters of 2020 are more pronounced (46% and 43%) and the numbers recover at the end of the year but do not exceed previous levels. Again, as for the outpatient population, this trend is more pronounced in children under five years of age (64% in the 2nd, 67% in the 3rd quarter).

**Table 2:** Quarterly rate ratios (RR), rate differences (ΔR) and p − values comparing the rates of 2020 with the combined rates of the years 2015 to 2019 for outpatients and malaria patients in health facilities of northern Ghana

	RR ΔR p − value	1st quarter	2nd quarter	3rd quarter	4th quarter
Outpatient department visits	All visits	0.93 − 269.0 0.202	0.80 − 745.3 < 0.001	0.73 − 1448.2 < 0.001	1.03 125.4 0.283
	All malaria	1.04 43.0 0.679	0.91 − 110.8 0.300	0.74 − 654.3 0.002	1.27 570.8 < 0.001
	Malaria children < 5 years	0.96 − 60.6 0.697	0.81 − 310.9 0.021	0.57 − 1614.5 < 0.001	1.19 589.5 0.005
	Malaria pregnant women	0.87 − 14.2 0.269	0.96 − 4.4 0.709	1.14 27.6 0.127	1.48 80.7 < 0.001
Inpatient department vistits	All malaria	0.79 − 36.8 0.139	0.54 − 65.1 0.008	0.57 − 139.7 < 0.001	0.94 −16.5 0.526
	Malaria children < 5 years	0.74 − 186.7 0.035	0.46 − 300.7 0.020	0.43 − 764.5 < 0.001	0.82 − 224.5 0.067

## Discussion

The Covid-19 pandemic has major consequences on the functioning of health services and direct and indirect effects on the burden of various diseases [29, 30]. In this paper, effects of the pandemic on malaria case numbers in health facilities of northern Ghana, a region highly endemic for malaria, are described.

In northern Ghana, a slight but significant decline was observed in malaria cases during the 2nd and 3rd quarter of 2020. This decline is even more significant considering that the period coincides with the rainy season in northern Ghana (May-November) when usually the majority of malaria cases are recorded. Cases only rebounded to the average levels of previous years at the end of 2020. This pattern was visible in both, outpatient and inpatient settings, but more pronounced in the hospitalized population. The same applies to children and adults, where reductions were also observed in both groups, but were more marked in children under five years of age. The marked decline in March/April 2020 can be explained by the extensive movement and gathering restrictions and early stay-at-home advices for COVID-19-like symptoms unless these get severe. Such measures have likely supported the hesitancy to visit health facilities during the pandemic, which in turn poses a major risk for developing severe malaria [13, 31]. The decline observed in March/April 2020 was even more remarkable in inpatients. This does not support our initial hypothesis, that in cases of more severe malaria manifestation, patients were still brought to health facilities and hospitalized, despite the pandemic. The findings from this analysis support the hypothesis, that the reported malaria burden in health facilities will shrink due to the effects of the COVID-19 pandemic in highly malaria-endemic countries [32]. They also support results of the WHO World Malaria Report [13], and they agree with results of similar studies from other SSA countries classified as highly endemic for malaria, such as Sierra Leone, Uganda and the Democratic Republic of the Congo [33–36].

The distinct decrease of OPD visits in the health facilities of northern Ghana in September 2020 could be explained by unusual heavy floods that started mid-August, which might have further complicated the access to health services. These floods have provided a favourable habitat for *Anopheles* mosquitoes, what could explain the observed increase of malaria cases in October 2020.

Malaria cases seen in health facilities among pregnant women show a different trend. After a decline in April 2020, cases have rebounded rapidly in this population and reached even higher levels compared to previous years. The most likely explanation of such an opposite trend would be the early hesitancy of pregnant women to visit health facilities. This is probably due to the fear of getting infected with COVID-19, combined with initial disruptions of the provision of IPTp to women in antenatal care (ANC) services as well as the disruption of routine distribution of ITNs [37]. The disrupted access to and delivery of ANC services is likely to explain the malaria case trend in April. However, without IPTp and ITNs, more women were at risk for malaria thereafter, which can explain the subsequent rise in malaria cases over the following months. Also, many pregnant women probably have sought the missed ANC with subsequent malaria diagnosis after the initial movement restrictions were lifted.

Ghana had already achieved high levels of ITN coverage, and no ITN mass campaign was planned for 2020 [13]. However, the routine distribution of ITNs, which is usually done in health facilities during ANC sessions and in primary schools, needed to be adapted to the COVID-19 measures, which included school closures from March 2020 until January 2021 [38, 39]. Also the seasonal malaria chemoprevention intervention for children and the annual indoor residual spraying of insecticides, which both require physical contact between the health workers and the community, needed to be modified [40, 41]. As another consequence of the COVID-19 pandemic, the provision of rapid diagnostic tests for malaria was fragile, which may have led to under-diagnosis of cases [42]. The main explanation for the lower number of malaria cases seen in health facilities was limited access to health facilities – public transportations were unavailable or unaffordable, and health facilities were closed or only provided reduced services [43, 44]. This is supported by findings from a study from Rwanda which showed that health facility visits for malaria decreased while community health services for malaria increased [43]. Finally, reports of hesitancy to visit health facilities due to fear of getting infected with COVID-19 were common [37, 42]. Last but not least, the malaria health care worker capacities were limited due to frequent reassignments to the control of COVID-19, to stigmatization or absence following quarantine, or to the development of COVID-19 disease or even death [14, 39, 45].

This study has strengths and limitations. A strength of the study is that the data represent a whole year of follow-up into the pandemic, which provides a more comprehensive picture of the effects compared to the previous studies with much shorter study periods. Also, the subgroup analysis of children under the age of five and pregnant women allows for a more complete picture. A major limitation is that the surveillance system itself may probably have been affected by the pandemic, producing a bias in the reported numbers. Massive underreporting could have falsified the observed trends and our conclusions. Moreover, it is not clear if the quality of surveillance data is fully comparable during the five years observed. The data from the Northern Region of Ghana may also not be representative for other malaria endemic areas in SSA, thus, the study has a limited external validity. Absenteeism in health facilities by people with malaria symptoms that have switched to self-medication or traditional medicine or that could not afford reaching official health care during the pandemic could also have had an albeit unknown effect on the malaria figures [46]. Especially in the first months of the pandemic, many people may have used malaria medication off-label to prevent and treat COVID-19 what may also have impacted the malaria situation [13].

## Conclusions

This study shows that the COVID-19 pandemic has been associated with reduced overall outpatient visits and reduced malaria cases reported from northern Ghana’s health facilities. Further data and qualitative explanations from Ghana and other SSA countries and in particular data from community-based studies are needed to fully judge the impact of the pandemic on the malaria situation on the African continent.

## Data Availability

The datasets used and/or analysed during the current study are available from the corresponding author on reasonable request.
